# Cognitive Control in Auditory Working Memory Is Enhanced in Musicians

**DOI:** 10.1371/journal.pone.0011120

**Published:** 2010-06-15

**Authors:** Karen Johanne Pallesen, Elvira Brattico, Christopher J. Bailey, Antti Korvenoja, Juha Koivisto, Albert Gjedde, Synnöve Carlson

**Affiliations:** 1 Neuroscience Unit, Institute of Biomedicine/Physiology, University of Helsinki, Helsinki, Finland; 2 Center of Functionally Integrative Neuroscience, Aarhus University, Aarhus, Denmark; 3 Pathophysiology and Experimental Tomography Center, Aarhus University Hospitals, Aarhus, Denmark; 4 Cognitive Brain Research Unit, Institute of Behavioral Sciences, University of Helsinki, Helsinki, Finland; 5 Finnish Center of Excellence in Interdisciplinary Music Research, University of Jyväskylä, Jyväskylä, Finland; 6 Medical Imaging Center, Helsinki University Central Hospital, Helsinki, Finland; 7 Medical School, University of Tampere, Tampere, Finland; 8 Brain Research Unit, Low Temperature Laboratory, Helsinki University of Technology, Espoo, Finland; Lund University, Sweden

## Abstract

Musical competence may confer cognitive advantages that extend beyond processing of familiar musical sounds. Behavioural evidence indicates a general enhancement of both working memory and attention in musicians. It is possible that musicians, due to their training, are better able to maintain focus on task-relevant stimuli, a skill which is crucial to working memory. We measured the blood oxygenation-level dependent (BOLD) activation signal in musicians and non-musicians during working memory of musical sounds to determine the relation among performance, musical competence and generally enhanced cognition. All participants easily distinguished the stimuli. We tested the hypothesis that musicians nonetheless would perform better, and that differential brain activity would mainly be present in cortical areas involved in cognitive control such as the lateral prefrontal cortex. The musicians performed better as reflected in reaction times and error rates. Musicians also had larger BOLD responses than non-musicians in neuronal networks that sustain attention and cognitive control, including regions of the lateral prefrontal cortex, lateral parietal cortex, insula, and putamen in the right hemisphere, and bilaterally in the posterior dorsal prefrontal cortex and anterior cingulate gyrus. The relationship between the task performance and the magnitude of the BOLD response was more positive in musicians than in non-musicians, particularly during the most difficult working memory task. The results confirm previous findings that neural activity increases during enhanced working memory performance. The results also suggest that superior working memory task performance in musicians rely on an enhanced ability to exert sustained cognitive control. This cognitive benefit in musicians may be a consequence of focused musical training.

## Introduction

Musical knowledge and skilfulness vary greatly across the population. This provides a basis for the study of how individual differences are reflected in brain activity during perceptive and cognitive processes. Not surprisingly, musical competence facilitates both sensory memory and conscious cognitive processing of musical sounds, reflected in enhanced brain activity [1], [2], [3], [4], [5], [6]. The increased neural activity is explained by stronger acoustic encoding of musical sounds and also by the representation of stimuli in terms of multiple codes that can be exploited automatically [7]. For example, musicians recall visual patterns of successive musical notes better than non-musicians, probably because of musicians' knowledge of sound-labels [8]. However, evidence also indicates that musicians benefit from enhanced domain-general cognitive processes, including enhanced mathematical, verbal, and non-verbal skills [9], [10], [11], and non-musical enhancement of working memory in musicians has repeatedly been demonstrated. For example, musicians were able to remember more words from a recently presented list than non-musicians [12] and enhanced verbal memory [13] followed from musical training in children. Musicians also had shorter reaction times than non-musicians in a non-musical visual attention task, indicating greater ability to focus attention [14]. Cognitive control is an important component of working memory and may be critical in enhanced WM task performance. The notion that musicians may exhibit generally enhanced cognitive control and abilities to focus has interest in an overall learning perspective, since it has been suggested that these benefits could develop as a consequence of musical training and subsequent transfer to other cognitive domains [9], [10]. Long-term training in cognitive tasks was generally associated with activity increases in the lateral PFC and parietal regions [15], regions that were also repeatedly linked to top-down” cognitive control mechanisms. Specifically, lateral PFC regions were functionally linked to cognitive control during demanding tasks [16], and posited to be responsible for superior task performance [17], [18]. Moreover, the magnitude of PFC activity and task-relevant adjustments in behavior was found to relate positively to activity in the anterior cingulate cortex (ACC) [19], [20], which, together with bordering sections of the medial PFC, is associated with monitoring response conflicts [21], and predicting error likelihood [22], hence serving a supporting role in the engagement of cognitive control. Brain regions involved in the optimization of task execution also includes anterior parts of the lateral PFC, associated with the ordering of sequences of stimuli [23], [24].

We now test the hypothesis that musicians' superior performance in a demanding working memory task with musical chords depends on increased recruitment of brain areas involved in cognitive control, rather than enhanced processing in auditory cortical areas. Hence, to place high demands on cognitive control, we used the n-back task where stimuli appearing in sequences must continuously be memorized and compared. The stimuli were designed to minimize advantages of musical competence such as accurate encoding and distinction of sound features, by spacing the three different chords to be memorized by entire octaves. However, advantages to task performance could also be conferred on musicians via automatically enhanced memory traces or activation of stimulus-associated cues, such as descriptive musical terms. Two groups of participants, musicians and non-musicians, performed low load 1-back (1B) and high load 2-back (2B) WM tasks as well as passive listening (PL) that did not require memorization, while their blood oxygenation-level dependent (BOLD) brain responses were measured with functional magnetic resonance imaging (fMRI). The 2B vs. 1B contrast is well suited to assess changes in brain activity related to WM load, while other factors are kept constant, and we therefore focused on this contrast. We predicted that musicians would both perform better in the two WM tasks and have associated stronger brain activation, despite limited advantage from specialized musical knowledge. In particular, stronger activation of “top-down” cognitive control mechanisms would be reflected in enhanced responses in the parietal cortex, lateral PFC regions and ACC.

## Materials and Methods

### Ethics Statement

The volunteers gave informed consent to the study, as approved by the ethics committee of the Helsinki University Central Hospital.

### Participants

We recruited 10 participants aged 22–31 years (mean age 25 years, 5 women) with minimal musical training, obtained exclusively as obligatory primary school education, and 11 classical musicians aged 21–34 years (mean age 28 years, 9 women), who were either students or graduates of the Sibelius Music Academy in Helsinki, Finland. All participants were right-handed and had no history of neurological disease or hearing deficit.

The non-musicians in the present study were previously studied as a separate group, with focus on task-related decreases during working memory [25]. We also previously compared non-musicians and musicians in their behavioural and brain responses to the three different musical chords (major, minor, dissonant). Significantly different brain responses to chord type were detected during the passive listening condition only, while not detectable during the working memory conditions [26]. Here, we will focus on the effect of WM load on brain responses in non-musicians and musicians.

### Stimuli

The stimuli were 9 sound combinations (chords) of “major”, “minor” and “dissonant” chord categories according to the Western tonal music theory, each spanning three frequency levels separated by an octave (frequency ratio 1∶2, in musical notation the lowest pitches of the chords were A3, A4 and A5). Each chord was produced with the grand-piano (piano 1) timbre of the Roland Sound Canvas SC-50 synthesizer with built-in samples, and played using the ENCORE software. The chords were edited by CoolEdit and SoundForge programs to be balanced in the loudness level and have the same duration (870 ms). The relatively long duration for single piano chords was chosen to maximize the emotional effects, which were studied separately [26]. The major chords consisted of A, C#, E, A, C#, and as such were characterized mostly by consonant intervals. The minor chords were made of A, C, E, A, C, thus including the minor third interval, considered in music theory as an imperfect consonance [27], [28]. The dissonant chords were made of A, Bb, G, Ab, C, including a minor second, the interval considered as the most dissonant in the literature [29], [30], [31], and several other dissonant intervals.

### Experimental conditions

The three experimental conditions included two n-back task conditions of memorizing the octaves of chords, an easy 1-back task (1B) and a difficult 2-back task (2B), in addition to a condition of passive listening to stimuli without cognitive evaluation (PL). After each stimulus, participants responded by pressing the left or right button of a response pad, with their right index or middle finger, respectively. Participants pressed the left button in the 1B task when the octave of the chord matched that of the previous trial, and in the 2B task when the octave matched the chord presented two trials back. In all other trials and the PL condition participants pressed the right button.

### Image acquisition

Both functional and structural MRI images were acquired on a Siemens Sonata 1.5T system using a birdcage head coil. T1-weighted images were obtained for co-registration purposes with an isotropic resolution of 1×1×1 mm^3^ [MPRAGE: TR = 1900ms, TE = 3.86ms, TI = 1100 ms, flip angle = 15°]. For functional imaging, a T2*-weighted gradient echo echo-planar imaging sequence was used [GE-EPI; TR = 3660 ms, TE = 40 ms, flip angle = 90°], with an in-plane resolution of 3.5×3.5 mm^2^ and a slice thickness of 4 mm. The entire brain and cerebellum were covered using 36 axial slices (no gaps). A single functional volume was acquired in 2760 ms, introducing a period of scanner silence (900 ms; no gradient noise) during which the stimuli were presented.

### Experimental procedure

Participants received written and oral instructions of the experimental conditions. Prior to the start of the experiment, they practiced the WM tasks and button presses in the scanner room. During practice we carefully observed and interacted with the participants to make sure that they felt confident in performing the tasks. Regarding the PL condition, participants were told to rest their mind from the task while still pressing a button after each stimulus. The chords were presented binaurally with MR-compatible headphones (Commander XG, Resonance Technology Inc.) and played at an intensity of approximately 80 dB, individually adjusted, so that participants could clearly hear the sounds and did not feel any related discomfort. In order to assure optimal perception of the sounds, stimulus presentation was interleaved with image acquisition. The experimental instructions (see below) were projected onto a screen at the foot of the MR patient bed, which the subjects viewed via a mirror attached to the head coil.

**Figure 1:**
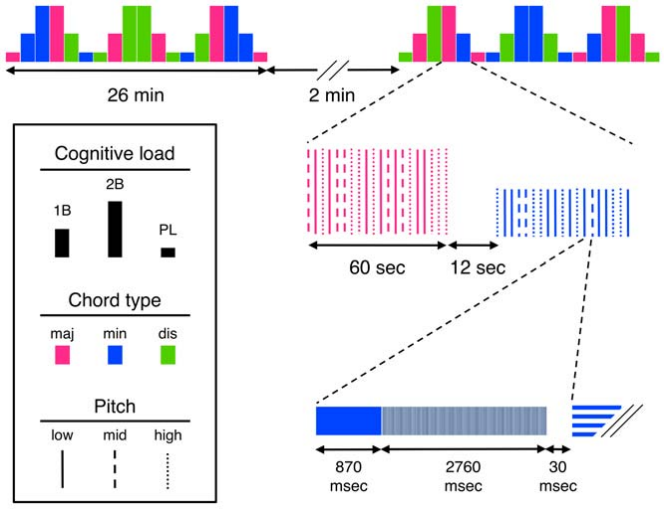
Experimental design. The experiment includes 36 blocks divided into two sessions (ca. 26 min each). A block consists of 20 trials and is defined by cognitive load (passive listening, 1B or 2B; indicated by column height), and chord type (major, minor or dissonant; indicated by color). The pitch height (low, medium or high) of each stimulus within a block, which is the memory item in the WM tasks, is indicated by line style. Experimental instructions are presented for 12 seconds between blocks on a screen viewed through a mirror. A single fMRI volume is acquired in 2760 msec (shaded area), allowing the stimuli (870 msec) to be presented during, and preceded by (30 msec), scanner silence (TR = 3660 msec).

The experimental design is illustrated in Figure 1. The experiment was divided into two sessions separated by a break of 2 min, during which the participants remained quietly at rest in the scanner but could move or close the eyes freely. A warning sound readied the subjects for the next session. Each session consisted of 18 blocks, each block defined by a task condition (PL, 1B or 2B) and a chord category (major, minor or dissonant); thus there were nine types of blocks: 1. PL (major), 2. 1B (major), 3. 2B (major), 4. PL (minor), 5. 1B (minor), 6. 2B (minor), 7. PL (dissonant), 8. 1B (dissonant), 9. 2B (dissonant). Each type of block was presented 4 times during the experiment in a counter-balanced design. An instruction screen was shown for 12 sec between the blocks to prepare the subject for the following task (“Passive Listening”, “1-Back”, or “2-Back”). During each block lasting 60 sec, 20 trials were presented, to which the subjects responded according to the task while fixating on a central cross on the screen. Each trial consisted of a sound presentation (870 msec), followed by image acquisition (2760 msec) and a brief silence (30 msec) before the next trial. Functional imaging lasted approximately 54 minutes, anatomical imaging about 7 minutes, resulting in a total time in the scanner of approximately one hour.

A questionnaire and a behavioural test followed the imaging part. The participants rated the task difficulty level as: 1. (very easy), 2. (easy), 3. (intermediate), 4. (difficult), and 5. (very difficult), and their level of alertness was rated at four different points (beginning, before break, after break and end) as alert, normal, tired or sleepy. Their memory strategies were evaluated by checking one or more of the following options: auditory rehearsal, verbal rehearsal, visual imagery, somatosensory imagery, movement, no specific strategy. In the behavioural test, participants rated the emotional connotation of each stimulus (major, minor and dissonant chords) on happy-sad and pleasant-unpleasant scales. Each scale had 11 values, from −5 to +5, −5 being the most negative rating and +5 the most positive rating, with zero as “neutral”. The chords were rated twice, presented in a randomized sequence.

### Behavioural data analysis

Statistical analysis of behavioural data was performed using the R project for statistical computing (www.r-project.org). Three-way repeated measures analysis of variance (ANOVA) was applied to the log-transformed reaction time (RT) data, with group membership as a between-groups factor, task condition and chord category as within-participant factors, and participant treated as a random effect. The test was implemented as a linear mixed-effects model (R function lme), after averaging the reaction times of individual participants within each experimental block. Accuracy of task performance was measured as the ratio (r) of incorrect responses over total (n) responses. The ratio was then transformed using the following formula: r′ = 2×pi×arcsin(p), where p = 1/(4×n), if r = 0; p = r , if 0<r<1; p = (n−1/4)/n, if r = 1. The arcsine transformation homogenized the variance of the binomial response variable (r). The transformed ratios (r′) were subjected to the same statistical test (three-way repeated measures ANOVA) as the log-transformed reaction times. The alpha-level used in all analyses is 0.05.

### Neuroimaging data analysis

All analysis of functional and anatomical MR data were carried out using the FMRIB Software Library (FSL, version 3.2b), Oxford Centre for Functional Magnetic Resonance Imaging of the Brain, UK (fmrib.ox.ac.uk/fsl/). Non-brain tissue was removed from the T1-weighted anatomical images using the Brain Extraction Tool (BET) [32]. The MNI/ICBM-152 average brain was used as the standard stereotaxic space template in group analyses [33], [34], [35]. Each individual's brain volume was co-registered to the template using affine transformations (12 degrees of freedom) estimated by FMRIB's Linear Registration Tool, FLIRT [36]. Functional data were processed, prior to normalization to stereotaxic space, using FMRIB's Expert Analysis Tool (FEAT). During the experiment, 896 volumes were collected, of which the first 5 volumes were discarded allowing T1 effects in the signal to saturate. Since the scanning had continued during the break between sessions there was no need to discard any images from the second session. The functional volumes were realigned to the midpoint of the experiment using rigid-body transformations (MCFLIRT [37]). Spatial smoothing was performed using a low-pass Gaussian filter with a FWHM of 8 mm. A piecewise linear temporal high-pass filter (longest period passed: 805 s) was used to remove low frequency components of the data. The GLM implementation of FEAT was used for model fitting of preprocessed data. The design matrix consisted of nine columns representing each of the possible blocks: permutations of memory load (2B, 1B or PL) and chord category (major, minor or dissonant). All columns were convolved with the “canonical” double-gamma hemodynamic response function. Autocorrelations in the model fit residuals were estimated and removed using the FILM prewhitening step in FEAT [38]. First-level contrast images between the 2B and 1B WM conditions were calculated separately within each chord category: 2B (major) vs. 1B (major), 2B (minor) vs. 1B (minor) and 2B (dissonant) vs. 1B (dissonant).

Group results were obtained with full mixed effects (ME) modelling, thus allowing generalization to the participant populations. In the present study, only the WM conditions were included in the group-level analysis, i.e. the PL condition was excluded. Higher level parameter estimates and the ME variance were estimated implicitly within FEAT using FMRIB's Local Analysis of Mixed Effects (FLAME) [39], [40]. The group mean 2B vs. 1B contrast was defined as a t-test of non-zero mean, where input contrast images were averaged across group, participant and chord category (we initially tested a model with chord type as an additional dependent variable; no effects of chord were found, consistent with our earlier findings in the WM conditions [25], [26]). The group difference (musicians vs. non-musicians) 2B vs. 1B contrast was similarly a t-test of differing means in the two groups. To allow for different first-level variances between the groups, they were modelled separately in FLAME. To control the type I error rate, Gaussian random field theory was applied to assign corrected significance levels to clusters of voxels surviving a threshold of Z>3.0; all imaging results presented in this paper are based on a cluster-level criterion of *p*<.05 [41], [42]. Tables of local Z-score maxima produced by FSL for each contrast were translated to anatomical names using a structural parcellation of the MNI single-subject brain [34], and the extended naming procedure described in [43].

### ROI-based regression analysis

To correlate task performance measures to the strength of brain responses, we conducted a region-of-interest (ROI) analysis on standard-space coordinates of group-dependently activated locations (cf. Results). A spherical ROI of 8 mm radius was created, centered on each coordinate, and resampled to each subject's functional data space using transformations estimated during intrasubject analysis. Mean percent BOLD signal changes were then extracted for the WM conditions, and used as the dependent measure in a regression of either the RT or “percent correct” rate (PC = 1−error rate) as the independent measure. Furthermore, load level, chord type and group membership were entered as categorical variables. Since none of the regions tested exhibited significant modulation of the regression by chord type (data not shown), this variable was removed from the final model, which then included the full 3-way load-by-group-by-BOLD interaction. Using this single model, we are able to test for significant differences between the slopes estimated for all four combinations of group and load. Regression analyses were performed in Stata release 10.1 (StataCorp, College Station, TX, USA).

## Results

### Behavioural results

**Figure 2:**
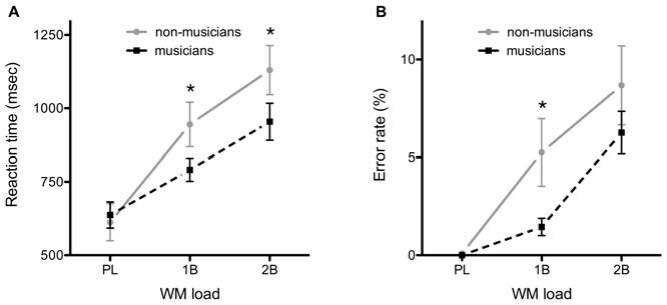
Effect of WM load on task performance measures as a function of group. A) Reaction times (RTs) increase as a function of load in both groups. In the WM conditions, the musicians respond faster than the non-musicians. B) Error rates also increase with increasing load in both groups. The musicians performed the WM tasks more accurately than the non-musicians. Asterisks (*) indicate significant group differences (*p*<.05).

The effect of WM load on reaction time was significant, F_2,144_ = 314.44, *p*<.0001. The grand mean ± SEM values for the PL, 1B and 2B conditions were 625.0±21.7 ms, 867.6±26.2 ms and 1041.7±31.8 ms, respectively (PL<1B: t_59_ = 10.94, *p*<.0001; 1B<2B: t_59_ = 12.01, *p*<.0001). Though no difference was observed on average between groups, F_1,18_ = 0.89, *p*>.05, the load-by-group interaction was significant, F_2,144_ = 18.80, *p*<.0001, illustrated in Figure 2A. The musicians responded faster than non-musicians in both the 1B (790.9±23.1 ms vs. 945.1±43.6 ms; t_44_ = 3.15, *p*<.005) and the 2B (954.1±36.6 ms vs. 1130.2±47.5 ms; t_54_ = 2.94, *p*<.001) conditions. There was no difference between groups in the PL condition (637.2±25.1 ms vs. 612.6±35.8 ms; t_52_ = 0.56, *p*>.05). The dissonant chords were associated with slightly faster responses (813.7±32.8 ms) than major (848.7±34.2 ms) and minor (851.7±37.5 ms) chords, but the main effect of chord type was not significant (F_2,144_ = 2.59, *p* = .078), and neither was the chord-by-group interaction (F_2,144_ = 0.06, *p*>.05). The remaining interaction terms were likewise non-significant: load-by-group, F_4,144_ = 0.54, *p*>.05, and load-by-chord-by-group, F_4,144_ = 0.12, *p*>.05.

The effect of WM load on response error rates, Figure 2B, was predictably strong (F_2,144_ = 161.29, *p*<.0001), with the PL condition error-free (0.0±0.0%), the 2B condition the most error-prone (8.7±0.8%), and the 1B condition between the two extremes (3.5±0.6%; PL<1B: t_59_ = 9.00, *p*<.0001; 1B<2B: t_59_ = 8.16, *p*<.0001). As in the case of RT, a significant load-by-group interaction was revealed for the error rates (F_2,144_ = 5.24, *p*<.01). Non-musicians made significantly more errors in the 1B condition than musicians, 5.3±1.0% vs. 1.6±0.3% (t_52_ = 3.51, *p*<.001), and more errors also in the 2B condition, 10.4±1.4% vs. 7.1±0.8% (t_51_ = 1.27, *p*>.05), but this difference did not reach statistical significance. No main effects on the error rate were found of either chord (F_2,144_ = 0.12, *p*>.05) or group (F_1,18_ = 2.66, *p*>.05). The interaction terms load-by-chord (F_4,144_ = 0.01, *p*>.05), chord-by-group (F_2,144_ = 0.27, *p*>.05) and load-by-chord-by-group (F_4,144_ = 0.08, *p*>.05), were all non-significant

**Figure 3:**
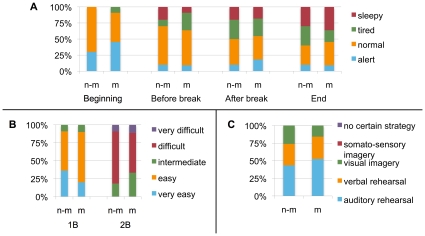
Subjective ratings. A) Task-difficulty, B) Alertness, and C) Employed task strategies. There were no marked differences between musicians (m) and non-musicians (n-m) in any of these subjective measures.

Participants' ratings of task difficulty levels, alertness levels and employed task strategies revealed a similarity between the groups. Regarding their experience of task difficulty, 90% of the participants rated the 1B task as “very easy” or “easy” (the remaining 2 participants, 1 musician and 1 non-musician, rated it as “intermediate) and 86% of the participants rated the 2B task as “difficult” or “intermediate” (the remaining 3 participants, 1 non-musician and 2 musicians, rated it as “very difficult”). The ratings were similar in the two groups and hence the task of memorizing the octaves was apparently experienced subjectively as equally feasible irrespective of musical competence (Figure 3B). The reported levels of alertness during the course of the experiment showed a development in alertness levels from alert to sleepy, which was also similar between groups (Figure 3A). The relative occurrence of the different task strategies was also similar in non-musicians and musicians (Figure 3C). The strategies “somato-sensory imagery”, “movement” and “no certain strategy” were not employed by any subjects. The majority, 8 musicians and 9 non-musicians, used several different strategies.

### Functional imaging results

Subject motion during scanning was corrected for using MCFLIRT [37], the output of which was used to calculate mean voxel displacements for each volume relative to the reference image and for each volume relative to the previously acquired volume (D_abs_ and D_rel_, respectively). The values (mean ± SEM) were: D_abs_ (non-musicians) = 1.04±.10 mm, D_abs_ (musicians) = 1.20±.09 mm, D_rel_ (non-musicians) = .05±.01 mm, and D_rel_ (musicians) = .06±.01 mm. The realignment data were not used in the subsequent GLM analysis, but manual inspection of the motion plots did not indicate stimulus-correlated motion (data not shown). Furthermore, in no subjects did the absolute or relative displacements exceed 1.9 mm and 0.15 mm, respectively. There were no statistically significant group differences between the displacements.

**Figure 4:**
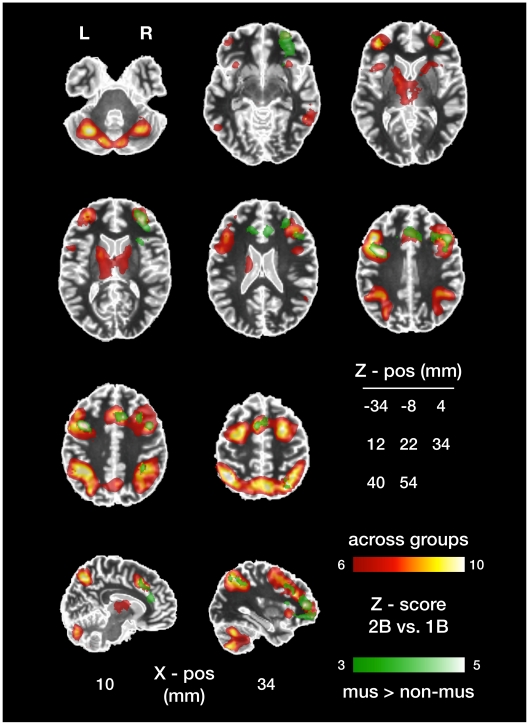
Working memory load-dependent brain responses differ between musicians and non-musicians. Regions in which the BOLD signal is significantly stronger during the 2B than the 1B task condition are shown in red/orange. In addition to this across-groups result, musicians' differential (2B vs. 1B) brain responses were significantly larger than non-musicians' in a subset of these regions shown in green. Both statistical maps are thresholded at Z>3.0 and corrected for multiple comparison at the cluster level (*p*<.05). The functional imaging results are overlaid on the MNI single-subject brain, which is displayed in the neurological orientation (left is left). The standard MNI space Z-coordinates (in mm) of the 8 axial slices are indicated in the “Z-pos” table.

The increased WM load in the 2B vs. 1B contrast manifested in an across-groups increase in brain responses. Significant clusters were localized bilaterally to the superior, middle and inferior frontal gyri, to the superior and inferior parietal lobules, and to precuneus, as illustrated in Figure 4 (red-orange colour scale) and listed in Table 1. The inferior, middle and superior temporal gyri, and orbital parts of the middle, inferior and superior frontal gyri also evidenced increased neural activity, as did several areas in the cerebellar hemispheres and vermis, the anterior/middle cingulate gyrus, thalamus, caudate nucleus, putamen, and insula.

**Table 1:** WM load-dependently activated regions: across groups average of the 2B vs. 1B contrast.

		Peak	Coordinates (MNI)				Peak	Coordinates (MNI)		
Brain region		Z score	x	y	z		Z score	x	y	z
*Angular gyrus*	L	8.88	−52	−50	34	R	15.9	34	−58	50
*Inferior parietal gyrus*	L	15.5	−36	−56	52	R	12.2	36	−54	48
*Supplementary motor area*	L	12.9	−8	10	50	R	11.1	2	14	50
*Superior parietal gyrus*		–	–	–	–	R	12.1	30	−70	54
*Triangular inferior frontal gyrus*	L	9.7	−42	42	0	R	11.5	44	30	24
*Middle frontal gyrus*	L	11.4	−40	50	2	R	11.1	30	18	44
*Orbital middle frontal gyrus*	L	11.4	−42	48	0	R	8.06	26	52	−4
*Superior frontal gyrus*	L	11.3	−30	0	66		–	–	–	–
*Operular inferior frontal gyrus*	L	9.51	−44	10	26	R	10.8	44	16	30
*Precentral gyrus*	L	10.6	−34	−4	64	R	8.74	40	4	44
*Inferior temporal gyrus*	L	7.12	−56	−60	−10	R	10.2	60	−44	−12
*Precuneus*	L	9.45	−6	−68	46	R	10.1	4	−66	46
*Cerebellum, Crus1*	L	10.1	−30	−64	−32	R	9.37	32	−62	−30
*Middle cingulate gyrus*		–	–	–	–	R	9.97	10	16	46
*Cerebellum, Crus2*	L	9.91	−34	−64	−40	R	8.62	12	−78	−30
*Cerebellum, 8*	L	7.21	−38	−52	−54	R	9.4	36	−66	−54
*Middle occipital gyrus*	L	9.29	−30	−72	40		–	–	–	–
*Medial superior frontal gyrus*	L	9.01	0	26	40		–	–	–	–
*Putamen*	L	8.96	−16	12	0	R	8.26	20	16	0
*Insula*	L	7.64	−42	16	6	R	8.68	34	28	−2
*Cerebellum, 7b*		–	–	–	–	R	8.63	26	−78	−52
*Anterior cingulate gyrus*	L	8.1	−6	30	32	R	5.76	12	28	22
*Caudate nucleus*	L	7.79	−10	8	10	R	6.55	14	−6	20
*Thalamus*	L	7.71	−10	−8	4	R	7.48	18	−14	12
*Orbital inferior frontal gyrus*		–	–	–	–	R	7.31	42	46	−4
*Inferior occipital gyrus*	L	6.87	−54	−66	−12		–	–	–	–
*Cerebellum, 9*	L	6.16	−22	−40	−42	R	6.59	8	−56	−54
*Cerebellum, 10*	L	6.29	−24	−36	−40		–	–	–	–
*Middle temporal gyrus*	L	6.08	−48	−52	8		–	–	–	–
*Orbital superior frontal gyrus*	L	6.04	−24	50	−6		–	–	–	–
*Cerebellum, 6*		–	–	–	–	R	5.96	6	−68	−24
*Vermis, 4/5*		–	–	–	–	R	5.89	2	−52	−24
*Superior temporal gyrus*	L	4.02	−64	−48	22		–	–	–	–
*Vermis, 9*		–	–	–	–	R	3.82	0	−56	−36
*Vermis, 8*		–	–	–	–	R	3.49	0	−64	−36

Nomenclature according to Tzourio-Mazoyer et al. (2002). Letters “L” and “R” refer to left and right hemisphere, respectively.

Musicians responded significantly more strongly than non-musicians to increased WM-load in a subset of the brain areas reported in the across-groups contrast, see Table 2 and Figure 4 (green colour scale). In no brain areas did non-musicians respond significantly more strongly than musicians. The increased responses in musicians were right-lateralized to the dorsomedial, frontopolar and orbital PFC regions and to the superior and inferior lateral parietal areas. Right-lateralized responses also appeared in the insula and putamen. We found bilateral responses in the posterior dorsal PFC (including medial parts of BA 6) and the anterior cingulate gyrus, and a left-sided response in the precentral gyrus. Neither the main effect of WM load nor the load-by-group interaction was modulated by chord type (data not shown); chord type information was excluded from the model on which the present results are based.

**Table 2:** Regions in which WM load-dependent activations (2B vs. 1B) were stronger in musicians than non-musicians.

		Peak	Coordinates (MNI)				Peak	Coordinates (MNI)		
Brain region		Z score	x	y	z		Z score	x	y	z
*Precentral gyrus*	L	6.49	−36	8	34		–	–	–	–
*Triangular inferior frontal gyrus*		–	–	–	–	R	5.35	50	22	30
*Middle frontal gyrus*		–	–	–	–	R	5.19	36	44	10
*Orbital middle fontal gyrus*		–	–	–	–	R	5.03	30	60	−4
*Middle cingulate gyrus*		–	–	–	–	R	4.98	12	20	40
*Orbital inferior frontal gyrus*		–	–	–	–	R	4.55	36	40	−8
*Supplementary motor area*	L	4.42	−6	8	56	R	3.48	4	12	52
*Inferior parietal gyrus*		–	–	–	–	R	4.09	38	−44	42
*Anterior cingulate gyrus*	L	3.98	−2	30	32	R	4.14	8	32	22
*Angular gyrus*		–	–	–	–	R	4	34	−58	50
*Insula*		–	–	–	–	R	3.68	34	20	14
*Putamen*		–	–	–	–	R	3.62	20	16	−4
*Superior parietal gyrus*		–	–	–	–	R	3.46	30	−70	58

Nomenclature according to Tzourio-Mazoyer et al. (2002). Letters “L” and “R” refer to left and right hemisphere, respectively.

### ROI-based regression results

In order to link the independent findings of enhanced performance and elevated BOLD signals in musicians, as compared to non-musicians, we extracted the percent BOLD signal change values for the WM task conditions from the group-dependently activated locations shown in Table 2. We used spherical ROIs with 8 mm radius, and performed the linear regression analysis described in Methods for each region separately. We focused attention on the slopes of the regression lines estimated for the load-by-group-by-BOLD model, i.e. the degree to which the brain-derived measure BOLD was correlated with the behavioural measures of percent correct responses and RT.

**Figure 5:**
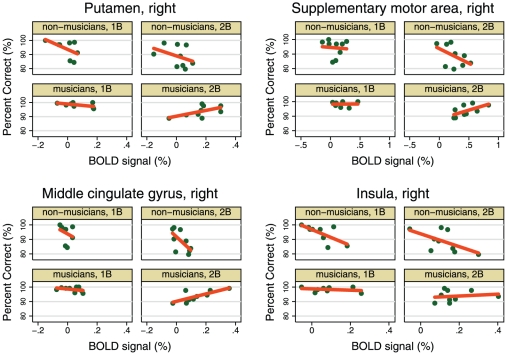
The strength of linear correlation between percent correct responses and the BOLD response differs significantly between musicians and non-musicians in the 2B task. The four regions illustrated are those that individually exhibit a significantly more positive linear slope in musicians than non-musicians in the 2B condition. This tendency, which is also present in the 1B condition, shows that by recruiting more brain resources during a WM task, musicians are able to sustain a higher performance level in face of the elevated cognitive demands. The green dots in each plot are measurements from individual subjects, and the orange line is the corresponding best-fitting regression line. NB: A single model was fit that was flexible enough to allow all four slopes to differ (see Methods for details); no R^2^-values are therefore given for the individual slopes. See Table 3 for a full account of the results and general tendencies for all regions.

The most consistent finding is that of a group difference in the measure linking successful task performance (PC) to BOLD signal strength in the 2B condition. Here, the slope of the musicians' regression line is more positive than the non-musicians, a tendency that reaches significance in the right putamen, the right supplementary motor cortex, the right insula and the right middle cingulate gyrus (Figure 5). We report all regression slopes and their group differences, for both WM task conditions, in Table 3. Each slope and difference value in Table 3 was tested against being zero, significant deviations from which are highlighted in bold typeface (*p*<.05). To emphasize the tendencies across brain regions, we calculated the median values for the slopes and their group differences, and applied the Wilcoxon signed-rank test to determine whether these medians were non-zero. In the 2B condition, the median correctness-to-BOLD slope was: significantly positive in musicians (7.21, *p*<.01), and significantly negative in non-musicians (−12.76, *p*<.05). Furthermore, the median group difference of slope was significantly larger than zero (17.36, *p*<.05). The results for the 1B task condition followed the same pattern, though they remained non-significant (cf. Table 3). No significant correlations were found between RT and the magnitude of the BOLD signal (data not shown).

**Table 3:** Summary of the ROI-based linear regression analysis of performance (Percent Correct) against BOLD signal amplitude, categorized by WM load (1B/2B) and group (musician/non-musician).

			1B			2B	
Region-of-interest, ROI		mus	non-mus	diff.	mus	non-mus	diff.
*Angular gyrus*	*R*	1.60	14.70	−13.10	7.21	20.04	−12.83
*Anterior cingulate gyrus*	*L*	−1.71	−2.37	0.66	8.19	−12.76	20.95
*Anterior cingulate gyrus*	*R*	−7.87	−76.49	68.63	15.52	−23.25	38.78
*Inferior parietal gyrus*	*R*	3.80	12.45	−8.65	9.71	18.13	−8.42
*Insula*	*R*	−4.50	**−54.93**	50.43	6.32	**−44.06**	**50.38**
*Middle cingulate gyrus*	*R*	−9.84	−56.60	46.76	25.62	**−94.26**	**119.88**
*Middle frontal gyrus*	*R*	−1.15	15.51	−16.66	−4.79	6.87	−11.65
*Orbital inferior frontal gyrus*	*R*	−6.04	−3.83	−2.21	−3.18	−15.01	11.83
*Orbital middle fontal gyrus*	*R*	−0.22	−11.03	10.81	−0.73	−8.00	7.27
*Precentral gyrus*	*L*	0.00	22.04	−22.05	5.82	1.24	4.58
*Putamen*	*R*	−9.82	−46.61	36.79	20.62	−34.62	**55.25**
*Superior parietal gyrus*	*R*	−0.72	−10.95	10.23	4.94	−21.66	26.60
*Supplementary motor area*	*L*	4.27	−2.73	7.00	12.87	−4.50	17.37
*Supplementary motor area*	*R*	0.23	−3.50	3.73	12.23	**−20.20**	**32.43**
*Triangular inferior frontal gyrus*	*R*	−1.30	3.59	−4.89	5.46	−6.76	12.22

		mus	non-mus	diff.	mus	non-mus	diff.
Median slope or difference		−1.15	−3.50	3.73	**7.21**	**−12.76**	**17.37**
P(H0: slope or difference = = 0)		*0.08*	*0.31*	*0.31*	***0.00***	***0.03***	***0.01***

The values above are the slopes of the estimated regression lines and their group differences for each region. Significantly non-zero slopes/differences are highlighted in bold typeface (*p*<.05). The median values, across ROIs, for each slope/difference are given separately, along with the *p*-values for the null-hypothesis (H0) of median zero (Wilcoxon signed-rank test). See text for a detailed description of the ROI definition, the regression model applied, and for an analysis of the results. Letters “L” and “R refer to the left and right hemisphere, respectively; see also Table 2.

## Discussion

In the current study, the influence of individual differences on WM was studied in a comparison of musicians and non-musicians who memorized musical chords in an easy (1B) and a difficult (2B) WM task. The behavioural data revealed that musicians performed better than non-musicians in the WM tasks, although the two groups rated the difficulty level of the tasks similarly. The musicians also had higher increases in BOLD brain responses than non-musicians as a function of WM load increase. While the load-dependent brain responses across both groups were bilateral (as in previous n-back WM studies [44], [45], [46], [47], [48]), this differential response pattern was mainly right-lateralized. Moreover, in the 2B task, musicians had a more positive correlation between WM task performance and BOLD signal amplitude, than non-musicians.

The across-groups results successfully reproduced the known relationship between working memory load and task performance [49], [50], [51] by showing that an increased WM-load leads to increases in both RT and the number of erroneous responses. The pattern of the load-dependent incremental brain activity included frequently reported “WM structures”, notably the PFC and posterior parietal cortex. Previous studies of visual verbal [44], [46], [52], [53], visual spatial [45], auditory spatial [47] and auditory verbal [54] n-back tasks all revealed load-dependent responses in the middle frontal gyrus (MFG), approximately corresponding to BAs 9, 46, and 10, superior parietal lobule (BA 7), inferior parietal lobule (BAs 39, 40) and posterior dorsal PFC (BAs 6, 8). The cerebellum also previously displayed load-dependent incremental activity in the few n-back studies that included this structure in the field of view [46], [53]. Recent theories relate the cerebellum to optimization of stimulus perception and manipulation during increased cognitive load [55]. Findings also suggest that decreased cerebellar activity is characteristic of skill learning [56], [57]. In our study there was no differences between the groups in the cerebellar responses, although the theory outlined above could suggest decreases in musicians/increases in non-musicians, due to decreased/increased recruitment of cerebellar optimization processes. The thalamus was similarly characterized by across-groups increased response, while no group difference was found. The thalamus was, to our knowledge not previously observed to respond to increasing WM load. However, thalamus activity was frequently observed during perception and cognition and has been functionally linked to attention [58], [59], [60], specifically the alerting component [61]. Since attention may increase during a demanding, compared to a less demanding, WM task, this could explain the current results. Other across-groups load-dependently activated brain areas include the cingulate gyrus [45], [47], [52], insula [44], [45], [47], and precuneus [45], [47], [62].

Previous attempts to systematically relate individual differences in working memory to the patterns of brain activity have led to equivocal results, as both increases and decreases in activity in similar brain regions were observed in relation to improved, impaired or even unchanged performance. In one study, a low error rate in a WM task related to increased activity in the left lateral PFC and parietal cortex [17]. In another, increased activity in the middle frontal gyrus and parietal cortices appeared to result from long-term training, independently of changes in performance [18]. However, local activity decreases in the lateral PFC and additional regions in association with WM training and improved performance was also reported [63], and it was suggested that activity increases in the dorsal PFC represents the effort invested in task performance [64]. On the other hand, it has been posited that increases in activity during repetition of the same WM task specifically relate to the prevention of automation by the demand to keep trial specific information active [18]. These studies suggest that increased activity occurs in areas that are critical to cognitive control [17]. The results of our study showed an effect of musical competence which was notably one-way: the magnitude of the BOLD responses was enhanced in musicians compared to non-musicians, whereas no enhanced responses were found in non-musicians compared to musicians. Hence our results show that enhanced WM performance is accompanied by enhanced brain activity. This is further in accordance with brain imaging studies in the musical domain that reported superior performance as well as increased brain activity in musicians compared to non-musicians. By comparing WM studies with respect to the time course of changes during training, it has been noted that activity decreases in the lateral PFC and other regions tend to be registered in relation to short-term training while increases seem to relate to long-term training [15]. Hence this would imply that different processes are pronounced at different times during the period of training, which would give rise to a variation in the observed patterns of brain activity. The activity increases observed in the current study may be viewed in the perspective of long-term training in musicians.

The proposition that musicians' superior performance depends on the magnitude of load-dependent BOLD responses receives supportive evidence from the ROI-based regression analysis. The assumed linear relationship between BOLD in the chosen ROIs and percent correct responses during the 2B task, was consistently more positive in musicians than in non-musicians (Table 3 and Figure 5), a tendency that reached significance in the right putamen, right insula, right supplementary motor cortex and the right middle cingulate gyrus. If, as we propose, the elevated BOLD signals during the high WM load (2B) task are interpreted as a manifestation of the participants' brains allocating more resources to the execution of the task, then the *less negative* BOLD-to-correctness relationship in musicians than non-musicians indicates that as a consequence of their efforts, musicians' performance *deteriorated less*. In other words, by allocating more resources, as reflected by the magnitude of the BOLD signal, to task execution, the musicians were better able to uphold task performance than non-musicians. Indeed, this interpretation is corroborated by the higher behavioural performance measures in musicians, than in non-musicians.

The group difference in the slope of the regression did not reach significance in the 1B task. This may be related to the observation that the 1B task was generally easy for all subjects, measured both subjectively (individual reports) and objectively (performance). The small spread of data points this implies will, independent of other considerations, lead to wider confidence intervals of the estimated linear regression parameters. That there were only two WM task difficulty levels, one rather easy and one rather difficult may be considered a weakness of the current experimental design that was not designed with regression analysis in mind. More difficulty levels and more subjects could potentially confirm the reported tendencies.

The enhanced WM performance of musicians in our study could involve both music-specific and more general cognitive processes. A previous study found that during a pitch memory task (contrasted with a motor control task), musicians had greater activations of the right planum temporale, right supramarginal gyrus, and superior parietal lobules, than non-musicians [65]. Noteworthy, this differential brain activity was present even when participants were matched on performance, suggesting that enhanced processing in these regions in musicians could be automatic. It was also found that (in a group of non-musicians) pitch WM training over a period of five days leads to enhanced responses in superior temporal brain regions including Heschl's gyrus [66].

Increased WM load in the present study did not enhance neuronal responses in auditory sensory regions. This may partly be due to the nature of the task, which did not involve fine-grained pitch comparisons such as those employed by Gaab et al. (2003), and hence there was little need for musicians to use their extensive music-specific processing capabilities. Another influential factor could be the nature of the contrast (2B vs. 1B) which may not be optimal to elicit activity changes in sensory cortical regions [47].

In the currently employed WM task musicians may on the other hand have benefited from superior cognitive skills that did not relate to musical stimulus processing per se [11], [14]. The nature of the n-back task partly supports this interpretation, since the increasing need for control during the temporary storage of information in correct serial order in the n-back task, especially when n>1, has been linked to the MFG [52], [67], and activity in the lateral PFC regions was more generally linked to the need for cognitive control during demanding tasks [16]. Cognitive control mediated by lateral PFC regions was previously mentioned as the mediator of superior task performance [17], [18]. We suggest that cognitive control may be a key to a unified interpretation of findings from studies of individual differences in an attempt to explain the variation in WM responses as a function of task performance. The hypothesis that musicians recruit more resources for cognitive control is also supported by our observation of greater activity in musicians in the anterior cingulate cortex (ACC), which, together with bordering sections of the medial PFC, has been assigned a central role in monitoring response conflicts [21], and predicting error likelihood [22]. Specifically, the magnitude of activity was found to predict both greater PFC activity and adjustments in behavior [19], [20], hence supporting a role of the ACC in the engagement of cognitive control. Cognitive control also serves to keep active in mind the rules and goals that are relevant in a certain context, functions that are associated with lateral PFC regions [68]. The anterior PFC (BA 10), which also was more active in musicians, may be related to the integration of subgoals during WM [69], and the distinction of target from non-target stimuli during recognition [70]. Enhanced load-dependent responses in musicians were also found in the posterior dorsal PFC (approximately BA 6). This observation may be related to recent findings that link this region to the ordering of stimuli in a sequence [23] and the binding of individual stimulus units into a sequence [24], processes that are essential in updating the stimulus sequence and assigning temporal order in the n-back task. Maintenance of ordinal position, an essential component of WM, may also rely on verbal coding [71]. The greater activity in the right hemisphere triangular part of the inferior frontal lobe (BA 45) in musicians is intriguing. Since verbal reports did not reveal any group differences in the strategies used to perform the n-back task it could indicate enhanced automatic processing of musical syntax [72], potentially influencing the strength of the WM representations.

According to one interpretation, cognitive benefits in musicians that appear independent of their highly developed auditory sensory capacity may have developed during musical training and transfer to other cognitive domains. A recent long-term study clearly shows that non-musical enhancement in cognitive tasks can result from musical training-induced brain plasticity [10]. By following both the structural brain development and the musical and general cognitive development of children who received music lessons it was found that whereas after 15 months there was no evidence of transfer of cognitive skills to non-musical domains [73], after at least three years of training children who received music lessons performed better in both vocabulary and nonverbal reasoning skills [10]. Hence, the length of the training period most likely indicates the appearance of changes in brain activity. The present results do not constitute a basis for causal inferences, as superior cognitive control could be present from birth to a higher extent in musicians than in non-musicians, and hence may partly have primed musicians in their successful choice of career. However, it makes some sense to assume that musical skills, rather than a well-developed ability to focus, could be the primary determinant in musicians' choice of career. Hence, we tentatively suggest that the development of cognitive control may benefit from focused musical training, and that this cognitive benefit is reflected in enhanced brain activity during demanding cognitive tasks of any type.

The right-lateralization of the differential load-dependent responses in musicians observed in the current study represents a new addition to our knowledge of lateralization patterns during processing of musical sound stimuli, since several studies of musical competence and brain activity documented a relative shift to the left hemisphere in musicians compared to non-musicians during tasks requiring melody recognition [74], spectral musical tasks [75], [76], [77], passive listening [78] and rhythm perception [79]. However, these lateralization shifts in music experts were mainly explained in terms of the neural processing of complex musical sound features in regions including the planum temporale, the superior temporal gyrus and PFC. The latter area was specifically associated with the processing of musical rules and violations of conventional chord successions [72], [80], [81]. The left-lateralization observed in musicians was related to an increased analytical approach representative of tasks that require special musical skills [82], including the relative left-lateralization of responses during pitch processing in planum temporale in individuals with absolute pitch [78], [83]. On the other hand, processing of pitch-related aspects of musical sounds consistently was linked to predominantly right-lateralized temporal and prefrontal networks in the brain, based on lesion studies [84], [85], [86], [87], [88], [89], [90], [91] and human brain imaging studies [92], [93]. The minimization of music-specific processing demands in the present study could be a main factor influencing the observed right-lateralization of WM processing of musical pitch. As most comparative studies that investigated the lateralization of music processing in the brain relied on sensory abilities to recognize and identify complex musical sounds, the interaction between stimulus properties, musical competence, and processing requirements needs further investigation.

In summary, the results of the current study suggest that musicians are capable of recruiting more brain resources to sustain cognitive control during a WM task with musical chords than are non-musicians, and in doing so are able to sustain a higher performance level despite the elevated cognitive demands. There were no strong indications in our results that music-specific processes played a role in the superior performance of musicians, hence supporting previous evidence that cognitive control may be generally enhanced in musicians. Superior cognitive control could represent a skill that is established during demanding musical training and transferred to other cognitive domains. This finding bears important implications for the use of music to stimulate cognition, such as the ability to focus in school-age children.
